# Pre-Existing HCV Variants Resistant to DAAs and Their Sensitivity to PegIFN/RBV in Chinese HCV Genotype 1b Patients

**DOI:** 10.1371/journal.pone.0165658

**Published:** 2016-11-03

**Authors:** Yu Zhang, Ying Cao, Renwen Zhang, Xiaxia Zhang, Haiying Lu, Chihong Wu, Na Huo, Xiaoyuan Xu

**Affiliations:** Department of Infectious Diseases, Peking University First Hospital, Beijing, China; Inserm U0152, UMR 5286, FRANCE

## Abstract

**Background:**

The efficacy of direct-acting antiviral agents (DAAs) could be attenuated by the presence of resistance-associated variants (RAVs). The aim of this study was to investigate the natural prevalence of RAVs among Chinese HCV genotype 1b patients and analyze the efficacy of pegylated interferon (PegIFN)/ribavirin (RBV) therapy in patients with and without RAVs at baseline.

**Methods:**

Direct sequencing of the HCV NS3, NS5A and NS5B regions was performed in baseline serum samples of 117 DAAs-naïve subjects infected with HCV genotype 1b. The efficacy of PegIFN/RBV therapy in patients with and without RAVs at baseline was analyzed by comparing the response rates between patients with RAVs and patients with wild type virus.

**Results:**

The incidence of RAVs was 8.00% (8/100) in the NS3 region (T54S, n = 1, 1.00%; R117H, n = 5, 5.00%; S122T, n = 1, 1.00%; S174F, n = 1, 1.00%), 29.91% (32/107) in the NS5A region (L28M, n = 12, 11.21%; R30Q, n = 10, 9.35%; L31M, n = 1, 0.93%; P58S, n = 4, 3.74%; Y93H, n = 8, 7.48%) and 98.15% (106/108) in the NS5B region (L159F, n = 1, 0.93%; C316N, n = 103, 95.37%; A421V, n = 6, 5.56%). The response rates to PegIFN/RBV treatment did not differ between patients with or without RAVs in the NS5A region.

**Conclusions:**

Pre-existing RAVs, including key RAVs, were detected in Chinese DAAs-naïve patients infected with HCV genotype 1b. IFN-based therapy could be a good option for patients with RAVs, especially key RAVs, at baseline.

## Introduction

Hepatitis C virus (HCV) infection is a major cause of chronic liver disease that can progress to cirrhosis and hepatocellular carcinoma. More than 185 million people have been infected with HCV globally, of whom 350, 000 die each year [1]. The most prevalent HCV subtype in China is genotype 1 [2,3]. The rapid development of direct acting antivirals (DAAs) has revolutionized chronic hepatitis C (CHC) therapy after the era of interferon (IFN). Currently, several clinical trials on DAAs are being carried out in China. In light of pending approval of DAAs in China, there is an urgency to better understand the presence of resistance associated variants (RAVs) and the impact of pegylated interferon (PegIFN)/ribavirin (RBV) treatment on patients that have them.

The efficacy of interferon-free DAAs-based therapy could be attenuated by the existence of RAVs, especially key RAVs. For example, only 39% of patients with baseline signature RAVs at NS5A-L31, NS5A-Y93 and NS3-D168 achieved a sustained virological response (SVR) after treatment with daclatasvir (an NS5A inhibitor) and asunaprevir (an NS3/4A protease inhibitor, PI) combination therapy, while the SVR rate was 92% in patients without these RAVs [4]. Pre-existing RAVs that are present in patients who are naïve to DAAs have been described in previous studies [5–8]. However, the prevalence of naturally occurring RAVs among Chinese HCV genotype 1b patients is not well known. A Japanese study demonstrated that Y93H RAV was more susceptible to IFN-based therapy than the Y93 wild type virus by performing both direct and deep sequencing at early time points after the start of treatment (within a week) [9]. IFN-based therapy may be one of the treatment options for patients that have RAVs. However, the efficacy of IFN-based therapy after early time points, such as SVR rates, in CHC patients with RAVs at baseline is not yet well understood.

The aim of this study was to explore the prevalence of naturally occurring RAVs in NS3, NS5A and NS5B regions and to analyze the efficacy of IFN-based therapy in Chinese CHC genotype 1b patients with and without RAVs at baseline.

## Patients and Methods

### Patients

Samples were obtained from 117 HCV genotype 1b patients from Peking University First Hospital between 2010 and 2014. None of these patients had been treated with DAAs prior to the commencement of this study. Among them, 71 patients were treated with PegIFN a-2a (Roche, Basel, Switzerland) at a dose of 180μg once per week and RBV (Meidakang, Sichuan, China) at a weight based dose of 15 mg/kg/d for 48 weeks. The HCV RNA loads were evaluated at weeks 4, 12, 24 and 48 after starting treatment and 24 weeks after the end of treatment. The diagnosis of CHC was made following EASL Recommendations on Treatment of Hepatitis C 2015 [10]. All the subjects were between 18 and 86 years old. Patients were excluded from the study if they were co-infected with hepatitis B virus or human immunodeficiency virus. HCV antibodies, HCV genotyping and serum HCV RNA loads were assessed according to the methods used in previous studies [11,12]. The presence of a genetic polymorphism in an SNP located near the IL28B gene (rs8099917) was determined with direct sequencing. Briefly, DNA was isolated from peripheral blood using QIAamp DNA Blood Mini Kit (Qiagen, Germany) and was amplified using Premix Taq (TAKARA, Japan) with the pairs of primers IL28B F 5’-TTGTCACTGTTCCTCCTTTTGTTT-3’ and IL28B R 5’- TGGGAGAATGCAAATGAGAGATA-3’. Written informed consent was obtained from all patients, and the research protocols were approved by the Medical Ethics Committee of Peking University People’s Hospital, which was the higher authority.

### Amplification and sequencing

Viral RNA was extracted from 140 μl of serum using QIAamp Viral RNA Mini Kits (Qiagen, Germany). The extracted RNA was reverse-transcribed and amplified using PrimeScript One Step RT-PCR Kit (TAKARA, Japan) with the primers NS3F1/NS3R1, NS5AF1/NS5AR1 and NS5BF1/NS5BR1. The targeted HCV genome was amplified by nested PCR using Premix Taq (TAKARA, Japan) with primers NS3F2/ NS3R2, NS5AF2/NS5AR2 and NS5BF2/NS5BR2 (Table 1). Primers shown in Table 1 were synthesized according to previously described methods [13,8].

**Table 1:** Amplification and Sequencing Primers for HCV NS3, NS5A and NS5B in Genotype 1b Patients.

Gene	Primers	Sequence
**NS3**	**NS3F1**	**GCCCGTCRTCTTCTCTGACATGG**
	**NS3R1**	**TTGTACCCTTGGGCTGCATA**
	**NS3F2**	**TCATCACCTGGGGGGCAGAC**
	**NS3R2**	**GTGCTCTTGCCGCTGCCAGT**
**NS5A**	**NS5AF1**	**GGATYAAYGARGACTGYTCYAC**
	**NS5AR1**	**GACCARGACCCGTCRCTGAGRT**
	**NS5AF2**	**GGGAYTGGATATGCACGGT**
	**NS5AR2**	**GGCATGGAGGARTAYGAC**
**NS5B**	**NS5BF1**	**CGYTGAGTCRTAYTCCTCCATGC**
	**NS5BR1**	**GGGCRCGAGACASGCTGTGATA**
	**NS5BF2**	**CTCAGYGACGGGTCYTGGTC**
	**NS5BR2**	**GCTGTGATATATGTCTCCCC**
	**NS5B Fwd Seq2**	**TGGGRGTHCGYGTRTGCGAG**
	**NS5B Rew Seq3**	**AGCATYGTGCAGTCCYGGAGC**

The direct sequencing of these PCR products was performed with an automatic sequencer (ABI 3730xl DNA Analyzer, ABI, USA). Sequencing primers that were used to complete the sequencing are shown in Table 1.

For analysis, nucleotide sequences were aligned with the HCV genotype 1b reference sequence (GenBank Accession No. AJ238799, http://www.ncbi.nlm.nih.gov/nuccore/AJ238799.1). The sequences were also submitted to the resistance database (http://hcv.bioinf.mpi-inf.mpg.de/index.php). As there has not been a universally accepted summary of DAAs resistance mutations, clinically relevant amino acid substitutions associated with resistance to DAAs were determined according to lists published in previous reports [14–21].

### Assessments of the efficacy of PegIFN/RBV

The efficacy of IFN-based therapy in Chinese CHC genotype 1b patients with RAVs at baseline was analyzed in 71 patients. Treatment responses at the defined time points were categorized as follows: (1) Rapid virological response (RVR): undetectable HCV-RNA(<15 IU/mL) at week 4; (2) Early virological response (EVR): a decrease in viral titer of >2-log10 compared with base-line values or negative results of HCV-RNA (<15 IU/mL) at week 12 of therapy (complete EVR, cEVR); (3) SVR: undetectable HCV-RNA (<15 IU/mL) 24 weeks after the completion of therapy [1,22]. The rates of RVR, EVR, cEVR and SVR were compared between patients with and without RAVs after stratification by IL28B genotype.

### Statistical analysis

Normally distributed continuous variables were expressed as the means±S.D.s, and non-normally distributed continuous data were expressed as medians (minimum, maximum). Categorical variables were summarized as a number (percentage). Frequency was compared between groups using the χ2 test or the Fisher exact test. The χ2 test was used when all expected frequencies were at least 1, otherwise the Fisher exact test was used. A *P* value of <0.05 was considered statistically significant. Statistical analysis were performed using the Statistical Package for the Social Sciences software version 16.0 (SPSS Inc, Chicago, IL, USA).

## Results

### General information about the patients

The mean age of these patients was 52.26±15.83, and 58 (49.57%) of the patients were male. The mean serum glutamic-pyruvic transaminase level was 61.88 IU/L and the median serum viral load was 6.36 log 10 IU/ml.

### Prevalence of NS3 PI resistance mutations

The NS3 gene was successfully sequenced in 100 of 117 samples (85.47%). Amino acid substitutions associated with the resistance to NS3 PIs were observed in 8 sequences (8.00%), including T54S (1.00%), R117H (5.00%), S122T (1.00%) and S174F (1.00%) (Table 2). Among them, T54S was known to confer low level resistance to first-generation PIs, including teleprevir and boceprevir, while R117H and S174F were considered to be possibly resistant to teleprevir and boceprevir. Other variants resistant to teleprevir and boceprevir were not observed. Only one sample was detected S122T which confers resistance to simeprevir and asunaprevir, second-generation PIs. Substitutions at the amino acid positions such as Q80, A156 and D168, which were also associated with resistance to second-generation PIs were not found. There were no combinations of resistance mutations.

**Table 2:** Prevalence of NS3 Protease Inhibitor Resistance Mutations in Genotype 1b Patients.

Residue	Resistance mutations	Drugs	Detected resistance mutations	Number of isolates (n = 100)(%)
**V36**	**A,M[15]**	**teleprevir, boceprevir**		
	**G[15]**	**teleprevir, asunaprevir**		
	**L,I[15]**	**teleprevir**		
**F43**	**V, I, S&[15]**	**simeprevir**		
**T54**	**A,S[15]**	**teleprevir, boceprevir**	**S**	**1(1.00)**
	**C,G[15]**	**boceprevir**		
**V55**	**A[15]**	**boceprevir**		
**Y56**	**H[15]**	**asunaprevir, paritaprevir**		
	**L[15]**	**asunaprevir**		
**Q80**	**K,R[15]**	**simeprevir, asunaprevir**		
**V107**	**I[15]**	**boceprevir**		
**R117**	H*	**teleprevir**	**H**	**5(5.00)**
**S122**	**I,T[15]**	**simeprevir, asunaprevir**	**T**	**1(1.00)**
	**A[15]**	**simeprevir**		
	**D,G,N[15]**	**asunaprevir**		
**R155**	**K[15]**	**teleprevir, boceprevir**		
	**K&[29]**	**grazoprevir**		
	**Q[15]**	**simeprevir, asunaprevir**		
	**C[15]**	**boceprevir**		
	**G[15]**	**asunaprevir**		
**A156**	**S[15]**	**teleprevir, boceprevir**		
	**T,V[15,14]**	**teleprevir, boceprevir, simeprevir**		
	**T&[29]**	**grazoprevir**		
	**F[15]**	**teleprevir**		
	**G[15]**	**simeprevir, vaniprevir**		
**D168**	**V[15]**	**simeprevir, asunaprevir, paritaprevir, vaniprevir**		
	**A[15]**	**simeprevir,paritaprevir,vaniprevir**		
	**K[15]**	**paritaprevir**		
	**E,F,H,T[15]**	**simeprevir,asunaprevir,vaniprevir**		
	**Y,G[15]**	**asunaprevir**		
	**Y&[29]**	**grazoprevir**		
**V170**	**A[15]**	**boceprevir, asunaprevir**		
	**T[15]**	**boceprevir**		
**S174**	F*	**teleprevir, boceprevir**	**F**	**1(1.00)**

* possibly resistant, it comes from the resistance database(http://hcv.bioinf.mpi-inf.mpg.de/index.php).

& mutations associated with resistance in vitro but not described in patients.

### Prevalence of NS5A inhibitor resistance mutations

The NS5A sequences of 107 samples were successfully obtained. Among them, 32 (29.91%) strains exhibited resistance mutations. The primary resistance mutations L31M and Y93H were detected in one (1/107, 0.93%) and eight (8/107, 7.48%) sequences, respectively. The L31M substitution confers 3-fold resistance to daclatasvir. The Y93H mutation is known to confer 24-fold resistance to daclatasvir, 77-fold resistance to ombitasvir and 1319-fold resistance to ledipasvir. Seven samples exhibited the L28M substitution which was known to confer resistance to ombitasvir in combination with L31F. R30Q was present in ten sequences (10/107, 9.35%), and four (4/107, 3.74%) sequences showed P58S, which were both considered to be secondary resistance mutations to daclatasvir that enhanced the resistance of primary mutations but did not confer resistance by themselves. In additon, P58S was possibly resistant to ombitasvir. Only two samples (2/107, 1.87%) exhibited more than one resistance mutation; one had R30Q+P58S, and the other had L28M+R30Q+Y93H. (Table 3)

**Table 3:** Prevalence of NS5A Inhibitor Resistance Mutations in Genotype 1b Patients.

Residue	Resistance mutations	Drugs	Detected resistance mutations	Number of isolates (N = 107)(%)
**L28**	**M [17,15]**	**daclatasvir, ombitasvir**	**M**	**12(11.21)**
	**T[15]**	**daclatasvir**		
**P29**	**S,X[15]**	**daclatasvir**		
**R30**	**G,H,P,Q#[19]**	**daclatasvir**	**Q**	**10(9.35)**
**L31**	**F [15,17]**	**daclatasvir, ombitasvir**	**M**	**1(0.93)**
	**M,V,L[15]**	**daclatasvir**		
**P32**	**L,X[15]**	**daclatasvir**		
**P58**	S*#[19]	**daclatasvir**	**S**	**4(3.74)**
**E62**	**D[15]**	**daclatasvir**		
**A92**	**K[15]**	**daclatasvir**		
**Y93**	**H[16–18,15]**	**ombitasvir,ledipasvir,daclatasvir**	**H**	**8(7.48)**
	**N[15]**	**daclatasvir**		
	**C[15]**	**Ledipasvir**		

* possibly resistant, it comes from the resistance database(http://hcv.bioinf.mpi-inf.mpg.de/index.php).

# secondary resistance mutations that increase the resistance of primary mutations but do not confer resistance by themselves

### Prevalence of NS5B inhibitor resistance mutations

Resistance mutations were detected in 106 strains (98.15%) out of the 108 successfully sequenced NS5B samples. Notablly, the majority of them were C316N, which was considered to possibly confer resistance to dasabuvir. Another resistance mutation, L159F, was detected in one strain. It was possibly resistant to sofosbuvir. A421V, which was associated with resistance to beclabuvir in vitro, was observed in 6 strains. C316Y, the high-level resistance mutation, which was known to confer 1569-fold resistance to dasabuvir, was not detected. S282T, L320F and V321A, which confered resistance to sofosbuvir in vitro, were not detected. P495L, resistant to beclabuvir in vitro, and S556G, associated with resistance to dasabuvir, were not found. Four strains exhibited more than one resistance mutation: one exhibited L159F+C316N+A421V, and the other two exhibited C316N+A421V. (Table 4)

**Table 4:** Prevalence of NS5B Polymerase Inhibitor Resistance Mutations in Genotype 1b Patients.

Residue	Resistance mutataions	Rrugs	Detected resistance mutations	Number of isolates (N = 108)(%)
**L159**	F*[20,15]	**sofosbuvir**	**F**	**1(0.93)**
**S282**	**T&[15]**	**sofosbuvir**		
**C316**	**Y[15]**	**dasabuvir**		
	N*[15]	**dasabuvir**	**N**	**103(95.37)**
**L320**	**F&[20,15]**	**sofosbuvir**		
**V321**	**A&[15]**	**sofosbuvir**		
**A421**	**V&[15]**	**beclabuvir**	**V**	**6(5.56)**
**P495**	**A,L,S&[15]**	**beclabuvir**		
**S556**	**G[15]**	**dasabuvir**		

* possibly resistant, it comes from the resistance database(http://hcv.bioinf.mpi-inf.mpg.de/index.php).

& mutations associated with resistance in vitro but not described in patients.

### Response to PegIFN/RBV in patients with and without RAVs

**Fig 1:**
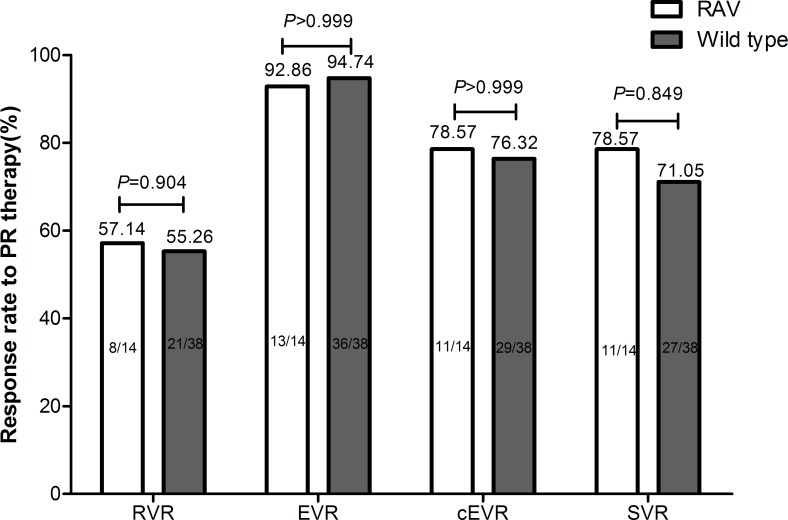
Virological responses to PR therapy in patients with and without RAVs in the NS5A region. Treatment response to PR such as RVR, EVR, complete EVR and SVR rates did not differ between patients with RAVs and patients with the wild type virus after stratification by IL28B genotype TT.

The response rates to PegIFN/RBV combination therapy (PR therapy) were analyzed in the 71 patients who received PR therapy during the follow-up. The NS5A sequence and IL28B (rs8099917) genotype were successfully obtained from 70 patients. Among them, 52 patients were of the IL28B (rs8099917) TT genotype. Among them, samples of 14 patients had detectable RAVs in the NS5A region at baseline. Treatment response to PR such as RVR, EVR, complete EVR and SVR rates did not differ between patients with RAVs and patients with wild type virus among patients with the IL28B (rs8099917) TT genotype. (Fig 1)

Since the number of IL28B (rs8099917) non-TT genotype patients was too small, the impact of RAVs on the response to PR therapy was not analyzed.

## Discussion

The HCV NS3/4A PIs teleprevir and boceprevir were first-generation DAAs approved for use in the clinic. They had a relatively low barrier to resistance compared with second-generation PIs such as simeprevir, asunaprevir and vaniprevir [23]. The prevalence of HCV NS3/4A PIs resistance mutations was 8.00%, which was similar to the prevalence of 10.7% reported in an Italian study [24]. Zeminian et al. reported that 18.9% of substitutions were involved in the resistance to PIs in Brazilian patients, which was higher than in the present study [25]. R117H and S174F, which were possibly resistant to first-generation PIs, made up the majority of the detected resistance mutations in this study. This finding was different from the results of other studies. For instance, in a Japanese study, Q80K/R and D168E/T which were resistant to second-generation PIs accounted for a relatively high proportion [7]. The difference may be due to the diversity of the epidemic strains of HCV in different areas. In the current study, amino acid substitutions that were resistant to teleprevir and boceprevir made up the majority. This finding indicated that second-generation PIs, which were more potent and had a better tolerance and a higher resistance barrier, will be more effective in Chinese CHC patients.

NS5A inhibitors have become important drugs and are included in most of the DAAs-based combination therapies for the treatment of CHC [10]. In the present study, Chinese genotype 1b CHC patients possessed a relatively high proportion (29.91%) of mutations involved in the resistance to NS5A inhibitors. Other studies have also reported the frequency of pre-existing resistance mutations [5,8]. For instance, a Tunisian report demonstrated a frequency of 16.2% of substitutions that conferred resistance to NS5A inhibitors in DAAs-naïve patients infected with HCV genotype 1b [5], which was lower than that of our study. L31M and Y93H were considered to be key resistance mutations; the presence of these mutations at baseline will significantly affect the outcome of DAAs-based antiviral therapy [4,23]. In our study, the prevalence of L31M and Y93H was 0.93% and 7.48%, respectively. The frequency of Y93H was not as high as that reported by Itakura (19.00%) [7], but was higher than that reported by an American study (3.77%) [26]. R30Q and P58S were found in 10 (9.35%) and 4 (3.74%) strains, respectively. Although these two variants are considered to be secondary resistance mutations, they should still be taken into consideration, since their presence will enhance the resistance of primary mutations [19]. The combination variants of L28M/L31F showed higher-level resistance to ombitasvir than L28M or L31F in their separate form [17]. In this study, L28M was detected without the presence of L31F.

NS5B polymerase inhibitors are potent agents with a high resistance barrier; as a result, they have been included in many recommended DAAs-based antiviral therapies[10]. The nucleotide analogue sofosbuvir and the non-nucleoside inhibitor dasabuvir are the two NS5B polymerase inhibitors that have currently been approved for the treatment of CHC. Resistance mutations to sofosbuvir mainly included L159F, S282T, L320F and V321A, which were known to be resistant in vitro only, but had not been confirmed in clinical trials. The combination variant L159F/L320F confers low-level resistance to mericitabine, a nucleotide polymerase inhibitor that has not been approved, and confers cross-resistance to sofosbuvir [20]. L159F was detected at a frequency of 0.93% without the presence of L320F in our research. Amino acid substitutions resistant to dasabuvir included C316N/Y and S556G. Christoph Sarrazin reported that C316N was observed at a frequency of 10.9 to 35.6% as a naturally occurring variant [27]. The frequency of C316N was significantly higher in our population, and was present in 95.37% of all tested samples. TheC316N variant may be more fit in Chinese genotype 1b CHC patients. C316N was possibly resistant to dasabuvir, which should be considered when dasabuvir is used to treat Chinese patients, due to the high prevalence of C316N in the Chinese population. C316Y and S556G confer high level resistance to dasabuvir, but they were not observed in our study. A421V was the amino acid substitution resistant to beclabuvir in vitro but was not described in clinical research studies. A total of 5.56% of the tested strains exhibited A421V, the prevalence of which was similar to that reported by Larousse [28].

Naturally occurring variants that conferred resistance to NS5A inhibitors were detected in a relatively high proportion of Chinese HCV genotype 1b patients. Patients with RAVs at baseline would respond to NS5A inhibitors relatively poorly, especially if they had key resistance mutations, such as Y93H and L31M. There was no difference in response to PR therapy between patients with or without RAVs in the NS5A region. This finding is in accordance with the results of a Japanese research study that compared RVR and EVR rates to PR therapy between patients with and without RAVs at baseline [7]. However, Itakura’s study showed that the Y93H RAV was more susceptible to IFN-based therapy than the Y93 wild type virus through direct and deep sequencing during the first seven days after the start of IFN-based therapy, which indicated that patients with RAVs at baseline were easier to cure with IFN-based therapy [9]. This finding was different from the results of the present study. The aforementioned research only studied the conditions during the first seven days after the treatment. As the treatment continued, HCV RAVs may have become as fit as wild type virus during IFN-based therapy. In addition, although RAVs are more sensitive to IFN-based therapy, a small percentage of RAVs among a patient may not have been sufficient to influence the efficacy of IFN-based therapy. Further studies are still needed to explore potential mechanism. It is certain that RAVs cannot attenuate the efficacy of IFN-based therapy. IFN-based therapy may be a good option for CHC patients with RAVs at baseline who do not have a contraindication for IFN. Moreover, the cost-effectiveness of PegIFN/RBV should also be considered in developing countries.

There are several limitations in our study. First, only the naturally occurring resistance mutations in HCV genotype 1b patients were analyzed. Second, the sequence information was not determined by ultra-deep pyro-sequencing. The sequencing technology used was the Sanger method, so the presence of minority variants of < 20% cannot be excluded. Despite these limitations, this study is important in helping to raise awareness of naturally occurring resistance mutations among Chinese CHC patients.

In conclusion, naturally occurring resistance mutations to DAAs did exist in Chinese DAAs-naïve patients infected with HCV genotype 1b. Some key resistance mutations such as L31M and Y93H were detected in these patients. Further studies are still needed to better understand the effect of RAVs in the future. IFN-based therapy may be a good option for patients with RAVs, especially for patients with key RAVs at baseline.

## Supporting Information

S1 File Data set underlying the findings in this study. This is the data including the sequences and the clinical data.
(RAR)
